# Validation of a green and sensitive spectrofluorimetric method for determination of Bilastine and its application to pharmaceutical preparations, content uniformity test, and spiked human plasma

**DOI:** 10.1186/s13065-025-01622-y

**Published:** 2025-08-31

**Authors:** Ahmed S. Ahmed, Khalid M. Badr El-Din, Ahmed A. Khorshed, Sayed M. Derayea, Mohamed Oraby

**Affiliations:** 1 Department of Pharmaceutical Analytical Chemistry, Faculty of Pharmacy, Sohag University, Sohag, 82524 Egypt; 2 Department of Pharmaceutical Analytical Chemistry, Faculty of Pharmacy, Minia University, Minia, 61519 Egypt; 3 Department of Biomedical Engineering, University of Alberta, Edmonton, AB T6G 1H9 Canada

**Keywords:** Bilastine, Spectrofluorimetry, Pharmaceutical formulation, Content uniformity

## Abstract

Bilastine (BIL) is a new second-generation antihistaminic drug used for the management of urticaria and rhino-conjunctivitis symptoms. Herein, a spectrofluorimetric method for determining BIL is described. The method is very sensitive, simple, quick, and green. The suggested method depended on the measurement of the original fluorescence of BIL in 1.0 M sulfuric acid at an emission wavelength of 385 nm after an excitation at 272 nm. The method was evaluated by the International Council on Harmonization (ICH) requirements. The relationship between BIL concentrations and the fluorescence intensities was linear in a range of 10.0–500.0 ng mL^− 1^, and the correlation coefficient was 0.9999. The detection limit was 2.9 ng mL^− 1^ and the quantitation limit was 8.8 ng mL^− 1^. The suitable sensitivity and selectivity of the suggested method enabled its application successfully in analyzing BIL in pharmaceutical tablets without any interfering effect from their excipients and in spiked human plasma with appropriate recoveries from 95.72 to 97.24%. Additionally, the suggested method was utilized for content uniformity testing.

**Supplementary Information:**

The online version contains supplementary material available at 10.1186/s13065-025-01622-y.

## Introduction

**Fig. 1:**
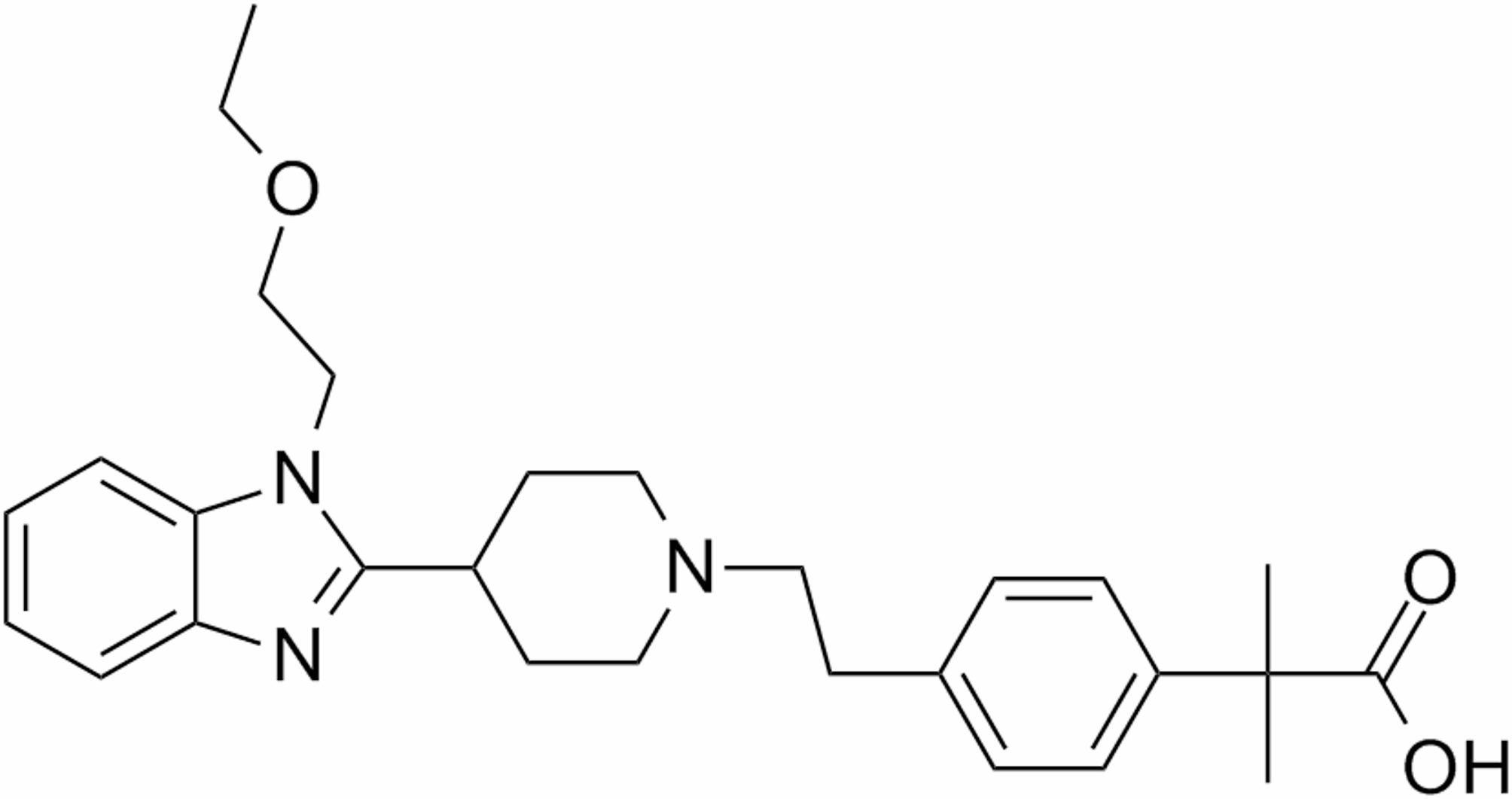
The chemical structure of BIL

Bilastine (BIL) is a new second-generation antihistaminic drug that is taken orally to treat the symptoms of urticaria and rhino-conjunctivitis, which can be seasonal or chronic [1]. The European Medicines Agency (EMA) approved BIL in September 2010 [2]. The recommended dose of BIL is 20 mg once daily for the management of urticaria and rhino-conjunctivitis symptoms. BIL has a high affinity for H_1_ receptors and has slight or no affinity for other receptors, including some histamine receptor subtypes, muscarinic and 5-HT receptors. So, it has no central nervous system (CNS) effects. BIL has a 6 and 3 fold higher affinity than fexofenadine and cetirizine, respectively [1, 3]. The chemical structure of BIL is 2-[4-(2-(4-(1-(2-ethoxyethyl)-1 H-benzimidazol-2-yl)piperidin-1-yl)ethyl)phenyl]-2-methylpropionic acid (Fig. 1) [4]. The available literature review revealed a variety of methods for analyzing BIL in bulk or pharmaceutical formulations. The methods that have been published for the analysis of BIL were spectrophotometric [5–7], fluorometric [8], HPLC [9–12], hydrophilic interaction liquid chromatographic method [4], HPTLC [13], Near-infrared spectroscopy [14], and electrochemical methods [15]. The HPLC technique needs a lot of extremely pure organic solvents, takes a long time to prepare samples, uses complicated apparatus, and, in certain situations, requires very expensive detectors. Moreover, the sensitivity of spectrophotometric methods is limited [16, 17].

Although direct spectrofluorimetric techniques have a great sensitivity, selectivity, and inherent simplicity [18], only one method for assaying BIL has been published [8]. in this reported method, the excitation and emission wavelengths were 272 and 298, respectively, which indicate high inner filter effect (IFE). IFE impedes fluorescence measurements by restricting fluorescence signal linear dependence to low sample concentrations [19, 20]. It has two types, Primary IFE (absorption of excitation light by the analyte or co-solutes prior to reaching the fluorophore), and secondary IFE (reabsorption of emitted light before detection). Because of the overlap between the excitation and emission wavelengths, the predominant IFE encountered in BIL is the secondary IFE. As a result, a straightforward, and easy spectrofluorimetric method with higher sensitivity for measurement of the antihistaminic drug BIL is considered essential. An easy, repeatable, and quick method is required for the analysis of BIL. The fluorescence characteristics of BIL in sulfuric acid media was investigated in order to develop the spectrofluorimetric method for BIL quantitation. Using sulfuric acid shifted the excitation and emission wavelengths to 272 and 385. As a result, inner filter effect (IFE) was diminished, and wide concentration range was determined. Furthermore, our method was extended to determine Bilastine in tablet content uniformity. The proposed method includes the use of water as a solvent which is the most eco-friendly solvent in analytical chemistry. The proposed method has the following advantages: a lower financial expense, low toxicity, simplicity, faster operation, and green [21, 22].

The suggested method was found to be compliant with the International Council on Harmonization (ICH) requirements [23], and it was successful in detecting BIL in bulk forms, pharmaceutical formulations, tablet content uniformity and spiked human plasma.

## Experimental

### Instrumentation

The spectrofluorimetric measurements were performed with JASCO FP-8350 spectrofluorometer (Hachioji, Tokyo, Japan). The instrument has a 150 W Xe-arc lamp and a PMT adjusted to a voltage of 400 V. Slits width for both emission and excitation monochromators were set to 5 nm, and the scanning rate was 1000 nm per min. To ensure the accuracy and reliability of fluorescence measurements, the spectrofluorometer wavelength was calibrated regularly using quinine sulfate solution (0.1 µg mL^-1^ in 0.1 M H₂SO₄), which has a known emission maximum at 450 nm upon excitation at 350 nm. To assess photostability, repeated measurements of standard solutions were conducted over time; fluorescence intensity deviations of less than ± 2% confirmed system stability during the analytical run. Distilled water was obtained using Aquatron water still a4000d (Cole-Parmer, Staffordshire, UK).

### Materials and reagents

BIL and Contrahistadin^®^ tablets containing 20 mg of BIL (B.N. H01702), were obtained from Global Advanced Pharmaceuticals (6th of October, Egypt). Spectroscopic grade methanol, ethanol, acetonitrile, tween, β-cyclodextrin (β-CD), sodium carboxymethyl cellulose (CMC Na), polyvinyl alcohol (PVA), citric acid, and perchloric acid were supplied from Merck (Darmstadt, Germany). Analytical grade dimethylformamide (DMF), sodium hydroxide, polyethylene glycol 400 (PEG 400), PEG 6000 were supplied from Fischer Scientific (Loughborough, U.K). Analytical grade acetone, sodium dodecyl sulfate (SDS), acetic acid, hydrochloric acid, sulfuric acid, nitric acid, and phosphoric acid were supplied by El Nasr Pharmaceutical Chemical Co. (Cairo, Egypt). Boric acid, citric acid, and phosphoric acid and sodium hydroxide were utilized for the preparation of Teorell - Stenhagen buffer solution pH (3–10) [24].

Human plasma was generously donated by Sohag University Hospital Blood Bank (Sohag, Egypt). It was kept frozen at − 20 °C until the analysis was done.

### Preparation of standard solution

Ten milligrams of BIL were dissolved in 250 mL distilled water to make stock standard drug solutions (40 µg mL^-1^). A portion of standard solution was diluted with distilled water to get working standard solutions that were used for calibration curve establishment.

### Procedure for general assay

Standard solutions of BIL in concentrations ranging from 0.1 to 5 µg mL^-1^ were transferred into 10 mL volumetric flasks, then 2 mL of 1 M sulfuric acid was added. After that, the flasks were filled to the final volume using distilled water and the contents were mixed thoroughly. The intensities of the fluorescence of the resulting solutions were monitored at 385 nm (λ_ex_ at 272 nm). A blank was processed using the previous steps except adding the BIL solution. Plotting the obtained values of the fluorescence versus the concentrations of BIL was carried out for construction of the calibration plot.

### Procedures for accuracy and precision

The method’s accuracy was evaluated using standard addition method. 1 mL of three different concentrations (500, 1500, and 2500 ng mL^-1^) of BIL standard solution were added to 1 mL previously analyzed BIL samples (500 ng mL^-1^) obtained from Contrahistadin^®^ 20 mg tablets. The analysis was performed for each concentration by applying the general analytical procedure in five replicates.

In order to evaluate the precision for the suggested approach, three concentrations spanning the BIL linearity range (100, 200, and 400 ng mL^-1^) were applied. A single day was used to measure intra-day precision, and three days were used to assess inter-day precision. The analysis was performed for each concentration by applying the general analytical procedure in five replicates.

### Procedure for selectivity

To evaluate the effects of different tablet additives used in tablet manufacturing and the selectivity of the existing method, 1mL of 1000 ng mL^-1^ BIL was mixed with 100,000 ng mL^-1^ of talc, zinc oxide, magnesium stearate, lactose, glucose, or starch. Then, the analysis was performed for each additive by applying the general analytical procedure in five replicates.

### Procedure for the analysis of BIL in tablet formulation

Ten Contrahistadin^®^ 20 mg tablets were precisely weighed and finely powdered. A portion of the fine powder containing 10.0 mg of BIL was moved to a 100 mL volumetric flask containing 30 mL of double-distilled water and the content was sonicated for 30 min. The flask was completed to the final volume with distilled water to get a solution of 100 µg mL^-1^ of BIL. After filtration using Whatman^®^ filter paper with pore size 11 μm and removing the initial part of the filtrate. Then 0.1 mL of the previous solution was transferred into 10 mL volumetric flask and completed to the final volume with distilled water to get a solution of 1000 ng mL^-1^ of BIL. Then 1 mL of the resultant solution was analyzed by applying the general analytical procedure in five replicates.

### Procedure for content uniformity test

The content uniformity (CU) test for BIL in tablet formulation was performed in accordance with USP requirements (Chap. 905) [25, 26]. A separate analysis of ten Contrahistadin^®^ 20 mg tablets was used for testing the uniformity of their contents. Each tablet was precisely weighed and finely powdered. A portion of the fine powder containing 10.0 mg of BIL was moved to a 100 mL volumetric flask containing 30 mL of double-distilled water and the content was sonicated for 30 min. The flask was completed to the final volume with distilled water to get a solution of 100 µg mL^-1^ of BIL. Then, testing the uniformity of their contents was completed using the procedure described under the analysis of pharmaceutical tablets for each individual tablet. Then, the acceptance value (AV) was calculated according to the following equation:

AV = KS+│M - X̅│[26].

where S represents the standard deviation, K represents the acceptability constant, M represents the reference value, and X̅ is the mean of each tablet content. If the AV was lower than or equivalent to the maximum permissible acceptance value (L1) 2.4, thus it was concluded that, the active ingredient quantity was uniform.

### Procedure for Estimation of BIL in spiked human plasma

To separate plasma proteins, centrifugation of 5 m mL of the blood sample was performed at 4000 rpm for 30 min. The obtained plasma was placed in Eppendorf’s tubes and kept at -20 °C. Into a clean tube, 1.0 mL of the stored plasma was transferred, 1.0 mL of BIL standard solution (final concentrations of 0.1–5 µg mL^-1^) was added, then 2.0 mL of acetonitrile was added as proteins precipitating agent. The tube was mixed by vortex for 60 s before being centrifuged for 10 min at 4000 rpm. The clear supernatant was moved to a clean tube and the general method procedure was followed. A blank experiment was treated similarly using distilled water instead of the standard drug solution.

## Results and discussion

**Fig. 2:**
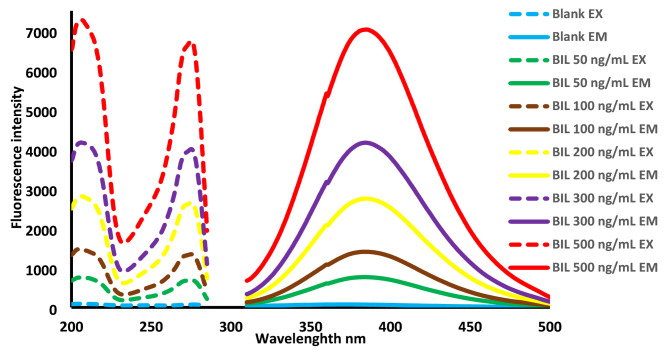
Excitation and emission spectra of sulfuric acid blank and 50, 100, 200, 300 and 500 ng mL^− 1^ BIL in sulfuric acid medium

The high fluorescence intensity of BIL is due to the presence of the benzimidazole ring in its structure. BIL shows strong native fluorescence at λ_em_ = 385 nm in the sulfuric acid medium (excitation at 272 nm), Fig. 2 which, decreased the IFE in the previous research Fig. S1. The effects of various experimental conditions on the fluorescence intensity of BIL were tested, and the best parameters for achieving the maximum fluorescence intensity were determined.

### Optimization of the experimental condition

#### Effect of buffers and pH modifier

**Fig. 3:**
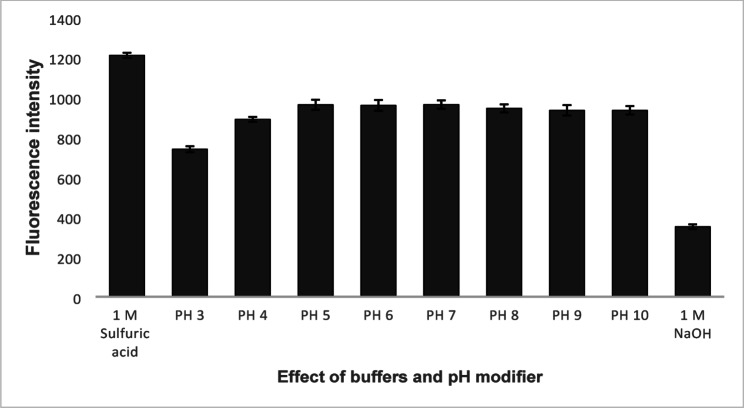
Effect of Teorell and Stenhagen pH, 1 M sulfuric acid and 1 M NaOH on relative fluorescence intensity of 100 ng mL^− 1^ BIL

**Fig. 4:**
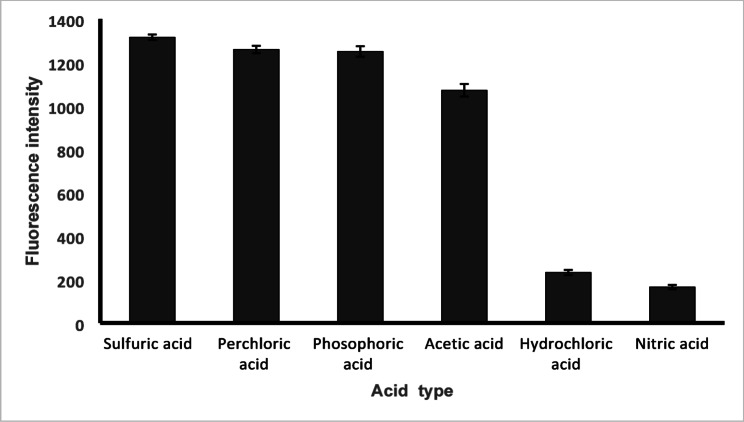
Effect of different acids on relative fluorescence intensity of 100 ng mL^− 1^ BIL

To enhance the native fluorescence of BIL, 1.0 mL Teorell-Stenhagen buffer solutions of varying pH (3.0–10.0) were examined in addition to 1.0 mL of 1 M H_2_SO_4_ or NaOH, Fig. 3. The best fluorescence intensity of BIL was obtained with 1 M H_2_SO_4_. Furthermore, it decreased the IFE. Different types of acids (1.0 M) were also investigated namely, sulfuric acid, perchloric acid, acetic acid, hydrochloric acid, phosphoric acid, and nitric acid, Fig. 4. The highest fluorescence intensity was obtained with 1 M sulfuric acid, so, it was used in the subsequent work as the pH modifier. As BIL contains basic functional groups (imidazole or piperidine moieties). When sulfuric acid was added, it protonated BIL, increasing its solubility in aqueous media and reducing its aggregation, protonation alters the electronic structure of BIL and reducing its absorbance in the excitation and emission wavelength ranges [27, 28]. Also, red shift in emission upon protonation as in neutral form, BIL has lone pairs on nitrogen that can conjugate with aromatic systems, leading to specific π→π* or n→π* transitions, and upon protonation, these lone pairs are no longer available, reducing electron density in the conjugated system. This stabilizes the lowest unoccupied molecular orbital (LUMO) more than the highest occupied molecular orbital (HOMO), resulting in smaller HOMO–LUMO gap, a lower energy required for excitation, and emission at longer wavelengths [27–29]. As a result, sulfuric acid minimizes the overlap between the absorption and emission spectra, thereby reducing the secondary IFE.

**Fig. 5:**
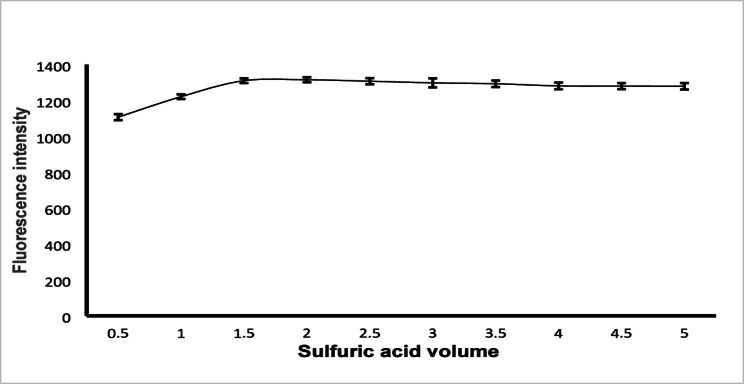
Effect of 1 M sulfuric acid volume on relative fluorescence intensity of 100 ng mL^− 1^ BIL

Finally, the effect of 1 M sulfuric acid volume was examined in the range from 0.5 to 5 mL, Fig. 5. The highest fluorescence intensity was obtained with the use of 2 mL of 1 M sulfuric acid.

#### Effect of different organized medium

**Fig. 6:**
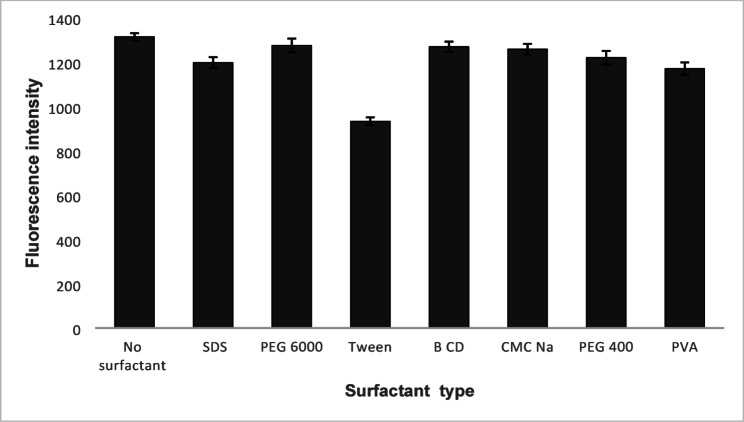
Effect of different surfactants on relative fluorescence intensity of 100 ng mL^− 1^ BIL

Various organized media were utilized in the study to enhance the fluorescence of the aqueous BIL solution. Anionic surfactant (SDS, 0.288% w/v), nonionic surfactant (PEG 6000, 1% w/v, PEG 400, 1% v/v and tween 80, 1% v/v, PVA, 1% w/v), anionic polysaccharide (CMCNa, 1% w/v) and macromolecules (β-CD, 1% w/v) were studied (Fig. 6). It was observed that; the studied substances did not enhance the fluorescence intensity of BIL; indeed, tween 80 significantly reduced the drug’s native fluorescence intensity. This may be attributed to fluorescence quenching via collisional interactions as the polyoxyethylene chains in tween can act as quenchers by forming transient complexes with the excited state of BIL, promoting nonradiative decay. Also, tween microenvironment alters the excited state of BIL, possibly facilitating dynamic quenching or internal conversion [27, 28]. As a result, no organized medium was used.

#### Effect of diluting solvent

**Fig. 7:**
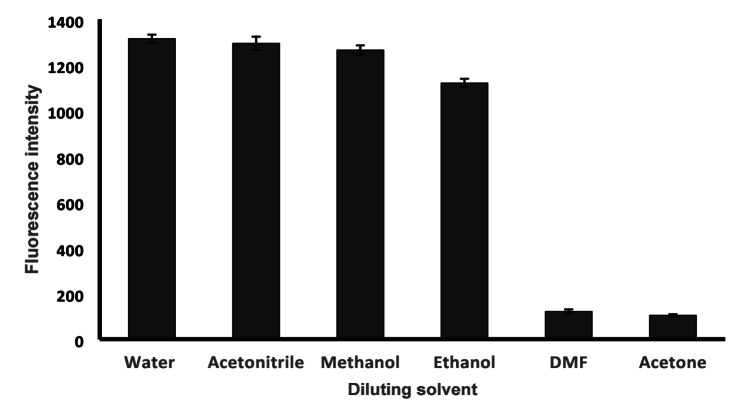
Effect of diluting solvent on relative fluorescence intensity of 100 ng mL^− 1^ BIL

Water, acetone, ethanol, methanol, dimethylformamide, and acetonitrile were studied to dilute BIL, Fig. 7. The greatest fluorescence intensity was obtained when water was the diluting solvent. The use of water is of a great advantage for the present work since water is eco-friendly, inexpensive, and readily available.

### Methods validation

ICH guidelines [23] were applied to evaluate and validate the proposed native fluorescence method.

### Linearity and range

The calibration curve was constructed by plotting various standard solution concentrations of BIL versus the fluorescence intensity. Linearity was achieved for the current method in concentrations ranging from 10 to 500 ng mL^-1^ in five replicates for each concentration and the correlation coefficient was 0.9999, indicating that; the suggested method has excellent linearity. The various analytical parameters were summarized in Table 1.

**Table 1:** The regression and validation parameters for the proposed method

Parameter	Proposed Method
Linear range (ng mL ^− 1^)	10–500
Slope	13.7764
SD of slope (S_b_)	0.0526
Intercept	-43.3058
SD of intercept (S_a_)	12.1247
Correlation Coefficient	0.9999
SD of residuals (S_y, x_)	27.3334
LOD (ng mL ^− 1^)	2.904
LOQ (ng mL^− 1^)	8.801

### Limits of detection and quantification

The method’s sensitivity was tested using the limits of detection (LOD) and limits of quantification (LOQ) calculations. The LOD and LOQ were estimated by applying the ICH guidelines equations LOD = 3.3 SD/b and LOQ = 10 SD/b (b is the slope and SD is the standard deviation of intercept). The found LOD was 2.9 ng mL^-1^, while the calculated LOQ was 8.8 ng mL^-1^, proving the current method is highly sensitive in the assay of BIL.

### Accuracy and precision

The accuracy of the provided fluorometric procedure has been examined using five replicates measurements of various BIL concentrations and using a standard addition method for each concentration. The obtained results demonstrated that the calculated values were highly agree with the actual values, indicating good precision of the suggested method, Table 2. Using the previously analyzed concentrations, the proposed fluorometric method has been tested for inter-and intra-day assay precision. To confirm intra-day precision, the experiment was repeated three times in one day (repeatability). To evaluate inter-day (intermediate) precision, the examined concentrations were measured across three days. As shown in Table 3, all the relative standard deviation values were below 2%, proving the excellent precision of the method.

**Table 2:** Accuracy of the proposed method using standard addition method

Amount taken from Contrahistadin^®^ (ng mL^− 1^)	Amount added (ng mL^− 1^)	Amount found (ng mL^− 1^)	% Recovery ± SD^a^
50	0	50.084	100.17 ± 0.59
50	50	100.121	100.12 ± 0.74
50	150	197. 897	98.95 ± 0.64
50	250	300.125	100.04 ± 0.42

^a^ Mean of five determinations

**Table 3:** Evaluation of the intra-day and inter-day precision for the proposed method

BIL Conc. ng mL^− 1^	Intra-day precision		Inter-day precision	
	Amount found (ng mL^− 1^)	% Recovery ± RSD ^a^	Amount found (ng mL^− 1^)	% Recovery ± RSD ^a^
100	99.976	99.98 ± 0.38	99.940	99.94 ± 1.14
200	199.930	99.96 ± 0.61	200.387	100.19 ± 0.94
400	401.652	100.41 ± 0.41	402.051	100.51 ± 0.96

^a^ Mean of five determinations

### Robustness

Upon introducing small variations in the parameters of the method, no effect was observed in the performance of the developed method. Fortunately, the method included only one parameter that could be examined, sulfuric acid volume. Minor variations in sulfuric acid volume had no apparent effect on the efficiency of the method. When 1.5 mL sulfuric acid was added, the recovery ± SD (mean of five determination) was found 98.23 ± 0.44 and When 2.5 mL sulfuric acid was added, the recovery ± SD were found 98.02 ± 0.51. Thus, the proposed fluorometric method was found to be robust.

### Selectivity

The influence of tablet excipients included in tablet manufacturing was explored, and the extent of their interference with the suggested approach was evaluated, to check the selectivity of the current method. Talc, zinc oxide, magnesium stearate, lactose, glucose and starch were tested. The results demonstrated the absence of any interfering effect from the examined excipients on the suggested method, as evidenced by the good recovery shown in Table 4.

**Table 4:** Evaluation of the selectivity for the proposed method

Substance added	Excipient added	BIL added	% Recovery ± SD ^a^	
	ng mL^− 1^	ng mL^− 1^		
Talk	10,000	100	99.03 ± 0.36	
Zinc oxide	10,000	100	100.60 ± 0.55	
Magnesium stearate	10,000	100	100.70 ± 0.66	
Starch	10,000	100	99.95 ± 0.44	
glucose	10,000	100	100.41 ± 0.48	
Lactose	10,000	100	100.12 ± 0.98	

^a^ Mean of five determination

### Pharmaceutical application

The suggested method was suitable for analyzing BIL in pharmaceutical dosage forms (Contrahistadin^®^ tablets). Table 5, shows that the percentage recoveries obtained were satisfactory, indicating that there is no matrix effect. For comparing the obtained results of the current method with the reported method results [5], the F-and student’s *t*-tests were used. Because the estimated values of both parameters were smaller than the tabulated values at the 95% confidence level, it was established that the accuracy and precision of the suggested method were not significantly differ from the reported method.

**Table 5:** Application of the proposedmethods for thedetermination of BIL inContrahistadin^®^ tablets (*n* = 5)

Parameters	Reported method	proposed method
% Recovery ^a^	99.40	99.16
Standard deviation, SD	1.20	0.86
Number of determinations	5	5
t-value ^a^		0.366
F-value ^a^		1.957

^a^ Tabulated value at 95% confidence limit; t = 2.306 and F = 6.338

### Application to content uniformity (CU) test

If the proportion of active elements in the tablet formulation units does not go beyond 25% of the entire weight of the tablet or if the content of the active constituent is less than 25 mg, it is advised that the CU of the tablet units should be investigated [25, 30]. For the first time, the spectrofluorometric method was utilized to track the CU of BIL in commercial tablets. Furthermore, the developed method had a very simple analytical process. As a result, the presented spectrofluorimetric method is ideal for this purpose. As presented in Table S1, the AV was lower than or equivalent to L1, thus it was concluded that, the active ingredient quantity was uniform in the studied pharmaceutical tablets.

The results obtained using the current spectrofluorimetric method for the analysis of Contrahistadin^®^ tablets (20 mg/tablet of BIL) were lesser than the L1 value. The spectrofluorimetric method has advantages of lower cost of chemicals and instrument, shorter analysis time and more eco-friendly than HPLC methods in measuring content uniformity of BIL tablets.

### Spiked human plasma application

It was reported that, BIL achieved its maximum plasma concentration (C_max_ = 220 ± 62 ng mL^-1^) 1.3–1.5 h after oral administration [1, 3]. BIL has a higher plasma protein binding ratio (84–90%) and approximately 95% of BIL was detected unchanged in plasma. BIL is not metabolized to significant extent in humans and is nearly removed from the body unchanged through both urine (33%) and feces (67%). Because the current method is highly sensitive, it was feasible to estimate BIL in biological fluids. In the analysis of spiked human plasma, the percentage recoveries were in the range of 95.72–97.24%. The results in Table S2, assured that; the suggested method was suitable for the precise assay of BIL in human plasma with no significant interference related to the matrix.

### Evaluation of method of greenness

Analysts wield considerable authority in safeguarding both individuals and the environment against the adverse effects of hazardous chemicals and the resultant waste generated within sectors such as chemicals and pharmaceuticals [31, 32]. It is imperative to regularly undertake the advancement and enhancement of green chemistry practices. Recent parameters like eco-scale scores and the Environmental Quality Methods Index have been employed to assess the environmental soundness of the analytical approach [33]. Our assessment of the method’s eco-friendliness was based on the eco-scale, which produces a numerical representation reflecting the penalties incurred during the research process. This quantifies the level of risk encountered, with higher scores indicating a more environmentally benign procedure. Notably, the developed method necessitated less than 0.1 kW/h of energy for a single sample’s processing, eliminating the need for heating or an extraction phase. Consequently, the proposed method received a commendable score of 95 on the eco-scale (see Table 6), unequivocally affirming its environmentally conscious nature.

**Table 6:** Evaluation of the greenness of the proposed spectrofluorometric method using the eco-scale score approach

Item	Parameter	Word sign	PP sign
**Technique**	Spectrofluorimetry	LSH	1
**Reagent(s)**	Non		0
**Solvent**	Water	LSH	1
**Heating**	No heating		0
**Temperature**	Room tempreture		0
**Cooling**	No cooling		0
**Energy (kW h per sample)**	≤ 0.1 KWh/sample		0
**Waste**	1–10 mL		3
**Occupational hazards**			0
**(TPPs)**			5
**Eco-scale total score**	= 100 - TPP		95

MSH is an abbreviation for the More severe hazard, LSH for the Less severe hazard, and TPPs for the Total penalty points

## Conclusion

The proposed spectrofluorometric method is very selective to determine BIL tablets’ formulations with no interference from their excipients. The presented work has the following advantages: it is sensitive, accurate, and precise when it comes to the analysis of the aforementioned antihistaminic drug in bulk, commercial tablet formulation. The simple procedure of the method enabled its application in tablet content uniformity. It was also able to analyze BIL in spiked human plasma due to the method’s good sensitivity (LOD = 2.9 ng mL^-1^). Furthermore, it is a time-saving method that eliminates the tedious steps for sample preparation or extraction. Due to its simplicity and sensitivity, this method is an excellent candidate for BIL quality control. The use of distilled water as a green solvent makes the proposed procedure a good alternative for conventional methods that use harmful organic solvents.

## Supplementary Information

Below is the link to the electronic supplementary material.

Supplementary Material 1

## Data Availability

All data generated or analyzed during this study are included in this published article.
