# Supplementary material for: Validation of a green and sensitive spectrofluorimetric method for determination of Bilastine and its application to pharmaceutical preparations, content uniformity test, and spiked human plasma
Source: BMC Chem. 2025 Aug 31;19(1):258. doi: 10.1186/s13065-025-01622-y (PMC12400534; doi:10.1186/s13065-025-01622-y)
Supplement: Supplementary file 1 — Supplementary Material 1 [file 13065_2025_1622_MOESM1_ESM.docx]

**Validation of a green and sensitive spectrofluorimetric method for determination of Bilastine and its application to pharmaceutical preparations, content uniformity test and spiked human plasma.**

Ahmed S. Ahmed ^a*^, Khalid M. Badr El-Din ^b^, Ahmed A. Khorshed ^a, c^, Sayed M. Derayea ^b^, Mohamed Oraby ^a^.

*^a^ Department of Pharmaceutical Analytical Chemistry, Faculty of Pharmacy, Sohag University, Sohag 82524, Egypt.*

*^b^ Department of Pharmaceutical Analytical Chemistry, Faculty of Pharmacy, Minia University, Minia 61519, Egypt.*

*^c^ Department of Biomedical Engineering, University of Alberta,* *Edmonton, AB, T6G 1H9, Canada.*

*Corresponding authors:* Ahmed S. Ahmed: **Ahmed.saad@pharm.sohag.edu.eg**

**Fig. S1:** The excitation and emission spectra of BIL without sulfuric acid.

**Table S1:** Application of the proposed methods for the content uniformity test of Contrahistadin^®^ tablets.

| **Tablet number** | **proposed method** |
| --- | --- |
| 1 | 98.52 |
| 2 | 100.19 |
| 3 | 98.23 |
| 4 | 103.97 |
| 5 | 101.21 |
| 6 | 102.73 |
| 7 | 103.31 |
| 8 | 98.52 |
| 9 | 98.02 |
| 10 | 97.44 |
| Mean X̅ | 100.22 |
| S | 2.43 |
| AV* | 6.57 |
| L1* | 15 |

^*^L1: maximum allowed acceptance value, AV: acceptance value.

**Table S2:** Determination of BIL in spiked human plasma by the proposed method.

| **Conc. level** | **% Recovery ± RSD** ^a^ | | |
| --- | --- | --- | --- |
| **ng mL^-1^** | **Intra-day precision** | **Inter-day precision** | |
| 25 | 96.78 ± 0.87 | 96.49 ± 1.42 | |
| 50 | 95.72 ± 0.91 | 95.90 ± 1.35 | |
| 100 | 97.24 ± 1.06 | 97.01 ± 1.80 | |
| ^a^ Mean of five determinations | | |  |
